# Seasonal patterns of taxonomic and functional beta diversity in submerged macrophytes at a fine scale

**DOI:** 10.1002/ece3.7811

**Published:** 2021-06-24

**Authors:** Hao Wang, Hui Fu, Zihao Wen, Changbo Yuan, Xiaolin Zhang, Leyi Ni, Te Cao

**Affiliations:** ^1^ State Key Laboratory of Freshwater Ecology and Biotechnology Institute of Hydrobiology Chinese Academy of Sciences Wuhan China; ^2^ University of Chinese Academy of Sciences Beijing China; ^3^ Ecology Department College of Bioscience & Biotechnology Hunan Provincial Key Laboratory of Rural Ecosystem Health in Dongting Lake Area Hunan Agricultural University Changsha China

**Keywords:** community composition, erhai, functional traits, seasonality, submerged macrophyte

## Abstract

Spatiotemporal variation in community composition is of considerable interest in ecology. However, few studies have focused on seasonal variation patterns in taxonomic and functional community composition at the fine scale. As such, we conducted seasonal high‐density sampling of the submerged macrophyte community in Hongshan Bay of Erhai Lake in China and used the generalized dissimilarity model (GDM) to evaluate the effects of environmental factors and geographic distance on taxonomic and functional beta diversity as well as corresponding turnover and nestedness components. At the fine scale, taxonomic turnover and nestedness as well as functional turnover and nestedness showed comparable contributions to corresponding taxonomic and functional beta diversity, with different importance across seasons. All taxonomic and functional dissimilarity metrics showed seasonal variation. Of note, taxonomic beta diversity was highest in summer and lowest in winter, while functional beta diversity showed the opposite pattern. Taxonomic and functional turnover showed similar change patterns as taxonomic and functional beta diversity. Taxonomic nestedness was low in summer and high in winter. Functional nestedness was also lower in summer. These results suggest that under extreme environmental conditions, both turnover and nestedness can exist at the fine scale and seasonal community composition patterns in submerged macrophytes should be considered. Future investigations on community assembly mechanisms should pay greater attention to long‐term dynamic characteristics and functional information.

## Graphical abstract

Both turnover and nestedness driven the spatial dissimilarity either from taxonomic or functional perspective in fine scale. Taxonomic and functional beta diversity, taxonomic and functional turnover, and taxonomic and functional nestedness all presented seasonal variations. Environmental variables rather than geographic distance dominated spatial variations of community assemblage in a fine scale.

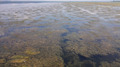

## 1 INTRODUCTION

How community assembly processes are driven by environmental factors and geographic distance remains a critical topic in ecology (Cottenie, 2005; Leibold et al., 2004; Socolar et al., 2016). The emergence of beta diversity concepts, representing spatiotemporal community dissimilarity from taxonomic or functional perspectives, has improved our understanding of biodiversity patterns (Baselga, 2012; Legendre & De Caceres, 2013; Legendre et al., 2009; Podani et al., 2013). To identify changes in internal community composition, beta diversity can be divided into turnover and nestedness components (Baselga, 2010, 2012; Villéger et al., 2013). In turnover, the loss of a species or trait is replaced by the gain of other species or traits, whereas in nestedness, species or traits that occur in one location are considered a subset of those from another location (Baselga, 2010; Villéger et al., 2013). Taxonomic and functional dissimilarity can characterize the different facets of community composition among sites. For example, communities may have higher species turnover but lower functional turnover as species may be replaced by those with similar traits (Fu, Yuan, Jeppesen, Ge, Li, et al., 2019). Changes in taxonomic and functional beta diversity aspects can also differ along environmental gradients for a particular community. For example, Bishop et al. (2015) found that with increasing elevational gradients, taxonomic community composition is driven by turnover while functional community structure is controlled by both turnover and nestedness patterns. Therefore, species‐based beta diversity alone may include limited information on the compositional changes among sites (Villeger et al., 2008, 2011). Incorporating functional traits into beta diversity indices could provide a more detailed understanding of biodiversity patterns and processes (Carvalho et al., 2020; Chun & Lee, 2017; Villéger et al., 2013).

However, the magnitude of beta diversity and relative importance of turnover and nestedness may depend on spatial scale (Declerck et al., 2011; Fu, Yuan, Jeppesen, Ge, Li, et al., 2019). Generally, both local environmental filtering and regional spatial processes (dispersal limitation with increasing geographic distance) are important factors of community composition changes among sites (Alahuhta et al., 2015; Legendre et al., 2009; Soininen et al., 2007). Theoretically, turnover patterns are influenced by environmental filtering, competition, and historical events, whereas physical barriers across large geographic distances may lead to nestedness (Alahuhta et al., 2017; Baselga, 2010; Legendre, 2014; Melo et al., 2009). For example, small species such as fish and zooplankton do not exhibit nestedness patterns at the fine scale as they can sufficiently and freely disperse, that is, show low dispersal limitation (Cottenie et al., 2001; Erős et al., 2012). In this situation, environmental heterogeneity will screen out species with specific adaptive capacity (He et al., 2019). Thus, species distribution patterns may mirror environmental gradients and turnover is prevalent (Baselga, 2012; Legendre, 2014). At the broad scale, species distribution patterns can include large geographic gradients (e.g., latitude, altitude) and dispersal barriers (Viana et al., 2015). Thus, community composition changes may originate from environmental filtering and dispersal limitations, with turnover and nestedness both important components of beta diversity (Declerck et al., 2011; Legendre, 2014). However, lake environments may change dramatically at the small scale (e.g., soil pH, total nitrogen (TN), and total phosphorus (TP)), thus filtering species less tolerant to extreme environments (e.g., water depth, water transparency), which, in turn, causes species nestedness among sites (Fu et al., 2020).

In freshwater lakes, submerged macrophytes are an important trophic level and play a vital role in determining ecosystem stability and services (Fu, Yuan, Jeppesen, Ge, Zou, et al., 2019; Fu et al., 2015, 2018; Scheffer et al., 1993). Thus, understanding the beta diversity patterns, including the turnover and nestedness components, of submerged macrophytes at different scales is essential. Previous studies on the spatial variation mechanisms of macrophyte assemblages have primarily focused on the larger scale. For example, Alahuhta et al. (2017) reported that turnover accounts for most beta diversity due to the variability in elevational range at the global scale. Fu, Yuan, Jeppesen, Ge, Li, et al. (2019) found that at the regional scale, different drivers contribute to structural (mainly turnover) and functional (mainly nestedness) beta diversity patterns in macrophyte species in Chinese lakes, and both species turnover and functional nestedness decrease with increasing water TP. Zhang et al. (2018) concluded that habitat loss and fishery intensity can impact the taxonomic and functional nestedness patterns of macrophytes, whereas water quality is a weaker driver at the regional scale. Excessive attention to large‐scale variation patterns of biodiversity will inevitably lead to a partial understanding of the generality of community organization and the drivers (e.g., water depth and transparency) that affect the distribution of submerged macrophytes on a fine scale will be ignored. Nonetheless, very few studies have examined taxonomic and functional beta diversity patterns in submerged macrophytes driven by environmental variables and geographic distance at the fine scale. In a small‐scale subtropical reservoir, Boschilia et al. (2016) found that landscape configurations, local environmental conditions, and biotic interactions cause high beta diversity (mainly species turnover). In addition, aquatic macrophyte communities exhibit greater seasonal variation in community composition than terrestrial plants. Rooney and Kalff (2000) found that macrophyte colonization depth increases in the warm season, resulting in greater productivity and distribution of submerged macrophyte communities. Therefore, integrating multiple sampling approaches will help provide a clearer understanding of the dynamic beta diversity patterns of submerged macrophytes.

Here, we examined seasonal patterns in submerged macrophyte beta diversity based on taxonomic and functional approaches at the fine scale (ca. 10 km^2^) (Hongshan Bay of Erhai Lake). We seasonally sampled 189 plots (200 × 200 m intervals between adjacent plots) evenly over the bay and measured taxonomic and functional beta diversities (Sørensen coefficient) and their turnover (Simpson coefficient) and nestedness components. Previous studies have found that strong winds and waves occur on Erhai Lake for most of the year (Chu et al., 2014), which transport propagules and thus weaken the effects of spatial processes on community structure. We used the generalized dissimilarity model (GDM) (Ferrier et al., 2007) to disentangle the role of environmental and spatial (i.e., geographic distance) processes in determining beta diversity patterns across the four seasons. Firstly, we hypothesized that turnover would be the main component of beta diversity (both taxonomic and functional), with nestedness only accounting for a small proportion. This is because wind and wave action in Erhai Lake may endow submerged macrophytes with a higher dispersal ability (i.e., low nestedness) (Chu et al., 2014), and thus, environmental variables will more strongly impact community structure (i.e., high turnover). Secondly, we hypothesized that beta diversity, including the turnover and nestedness components, would change seasonally. For example, beta diversity may be low in summer and high in winter regardless of taxonomic or functional beta diversity. This is because submerged macrophytes thrive in summer and species ecological amplitude is broader, which may result in a more homogeneous community structure in the bay (i.e., low taxonomic and functional beta diversity). In contrast, species habitat ranges may contract in winter, with only a few tolerant species able to survive the extreme environmental conditions, thus leading to a divergent community structure in the bay (i.e., high taxonomic and functional beta diversity). Thirdly, we hypothesized that environmental variables would be more important than geographic distance for taxonomic and functional beta diversity because of obvious environmental gradients (e.g., water depth) in the bay and weakened effects of geographic distance caused by strong wind and wave action in Erhai Lake (Wang et al., 2020).

## 2 MATERIALS AND METHODS

### 2.1 Study area

**FIGURE 1:**
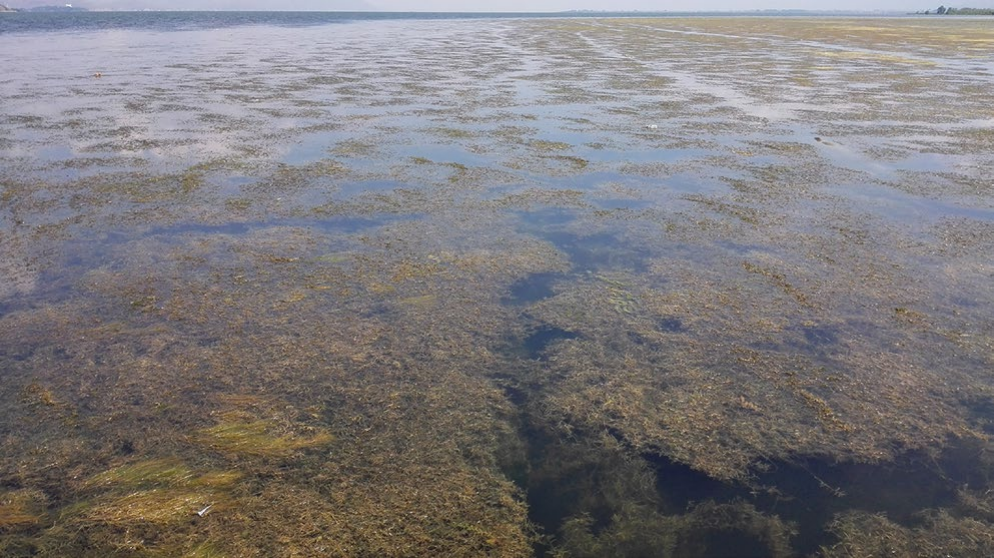
Submerged macrophyte community in Hongshan Bay, Erhai Lake

**FIGURE 2:**
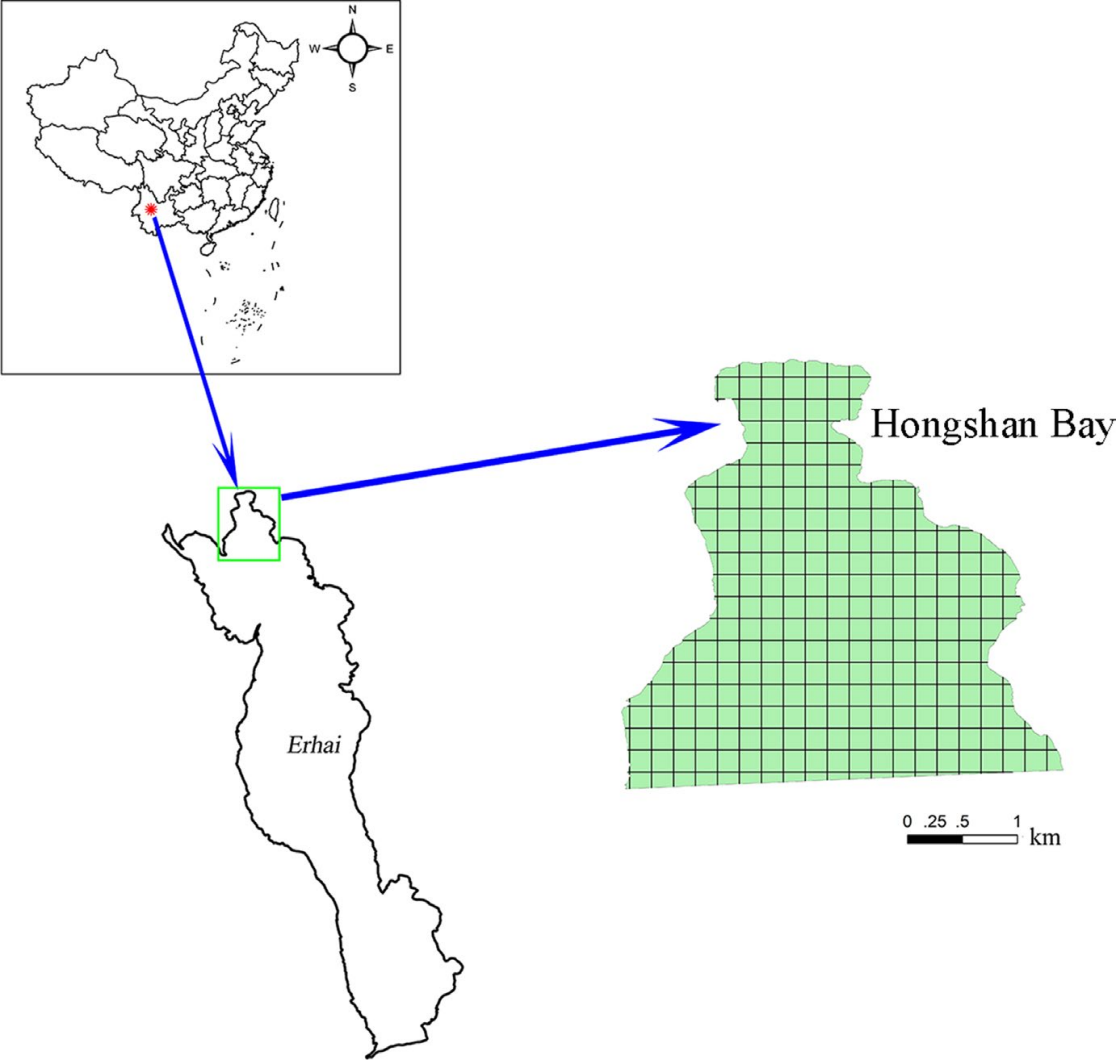
Location of sampling areas (Hongshan Bay) and distribution of sampling sites (grid intersections used for sampling)

The study was carried out in the northern Hongshan Bay of Erhai Lake (25°52′N, 100°06′E), Yunnan Province, China. Erhai Lake belongs to the northern subtropical climate, with a rainy season between May and October and a harsh dry period between November and the following April (Fu et al., 2013). Hongshan Bay is located at the northern end of Erhai Lake, with a maximum depth of 10 m and an area of 10 km^2^. In recent decades, there has been an increase in the external nutrient input in the lake with the development of surrounding cities. Thus, the water quality of Erhai Lake has changed from an oligotrophic state to a mesotrophic state (Lin et al., 2020), which has, in turn, lead to a change in the submerged macrophyte community from *Potamogeton maackianus* dominance to *Ceratophyllum demersum* and *P*. *maackianus* codominance (He et al., 2015) (Figure 1). The location of Erhai Lake and distribution of sampling sites are shown in Figure 2.

### 2.2 Data collection

The submerged macrophyte communities were sampled seasonally in 189 evenly sampling sites (mean intervals between adjacent plots of 200 × 200 m) across the bay. Sampling time occurred in April, July, and October 2018 and in January 2019, corresponding to spring, summer, autumn, and winter, respectively. In each survey, GPS coordinates were recorded using a GPS map 60CSx receiver at each site to calculate spatial variables. The submerged macrophyte communities were sampled three times at each sampling site with a rotatable reaping hook (area = 0.2 m^2^). Plants were washed and identified to species. In addition, TN, TP, chlorophyll‐a (Chl.a), water transparency (*SD*), and water depth (WD) were determined at each site as environment variables, as per Wang et al. (2020).

Based on our previous research, we measured 12 functional trait indices for the submerged macrophytes, which are widely used to analyze environmental adaptation (Fu et al., 2012; Fu, Zhong, Yuan, Ni, et al., 2014, Fu et al., 2017), including specific leaf area (SLA), relative growth rate (RGR), leaf area ratio (LAR), leaf mass ratio (LMR), shoot height, stem diameter, leaf dry mass content, lamina thickness, stem dry mass content, flowering duration, ramet size, and leaf carbon/nitrogen (C/N) ratio.

### 2.3 Data analysis

We used three complementary steps to investigate seasonal patterns in taxonomic and functional beta diversity of the submerged macrophytes. Firstly, taxonomic beta diversity (TBD) of submersed macrophytes was calculated using pairwise‐site Sørensen coefficients (β_sor_) based on presence–absence species data (Baselga, 2010; Baselga & Orme, 2012). Given that species turnover (β_sim_) and nestedness (β_sne_) combine to equal beta diversity (dissimilarity) and similarity equals 1 − dissimilarity, we used a triangular graph to describe the distribution of turnover and nestedness, that is, similarity = (1 − beta diversity), according to Legendre (2014). Each side of the triangle represents one of the three components. For functional beta diversity (FBD), we used the Sørensen coefficient, as suggested by Villéger et al. (2013): 1) We calculated the functional distance matrix among species with original trait data using Gower's distance (Gower, 1971); 2) principal coordinate analysis (PCoA) was used to create replaceable functional axes from the functional distance matrix, and we selected the first two axes as synthetic functional traits of submerged macrophytes because of long computation times; 3) finally, we used the “*functional.beta.pair*” function from the R package betapart (Villéger et al., 2013) to calculate the three functional dissimilarity indices: that is, beta diversity, turnover, and nestedness by combining species data and synthetic functional traits. We also used the triangular diagram mentioned above to represent functional beta diversity as well as its turnover and nestedness components. The relative importance of turnover and nestedness components for taxonomic and functional beta diversity was examined in each season using Wilcoxon signed‐ranks tests. The differences in beta diversity, turnover, and nestedness for taxonomic and functional facets across seasons were compared by Kruskal–Wallis post hoc comparisons.

Secondly, we also explored the effects of environmental factors and geographic distance on taxonomic and functional species assemblages. We used environmental factors, geographic distance, or both as the explanatory variables and beta diversity, turnover, and nestedness as the response variables to construct the GDM. As a nonlinear statistical approach, the GDM can explain spatial variation in community composition better than linear methods (Fitzpatrick et al., 2013; Glassman et al., 2017). The percentages of pure environmental and geographic distance contributing to the total explained deviance were calculated as the “pure” effect of environment and geographic distance for beta diversity, turnover, and nestedness (Fitzpatrick et al., 2013; Fu et al., 2020).

Thirdly, we used the GDM, which applies maximum‐likelihood estimation and flexible I‐splines, to evaluate the effects of specific variables (e.g., TN, TP). The maximum height of each I‐spline represented the importance of a specific variable on beta diversity, turnover, and nestedness, while holding all other variables constant (Ferrier et al., 2007). The GDM was constructed using the “*gdm*” function in R with the “gdm” package (Fitzpatrick et al., 2020). All statistical tests were performed in R v3.60 (R Core Team, 2019).

## 3 RESULTS

### 3.1 Relative importance of turnover and nestedness in taxonomic and functional beta diversity

**FIGURE 3:**
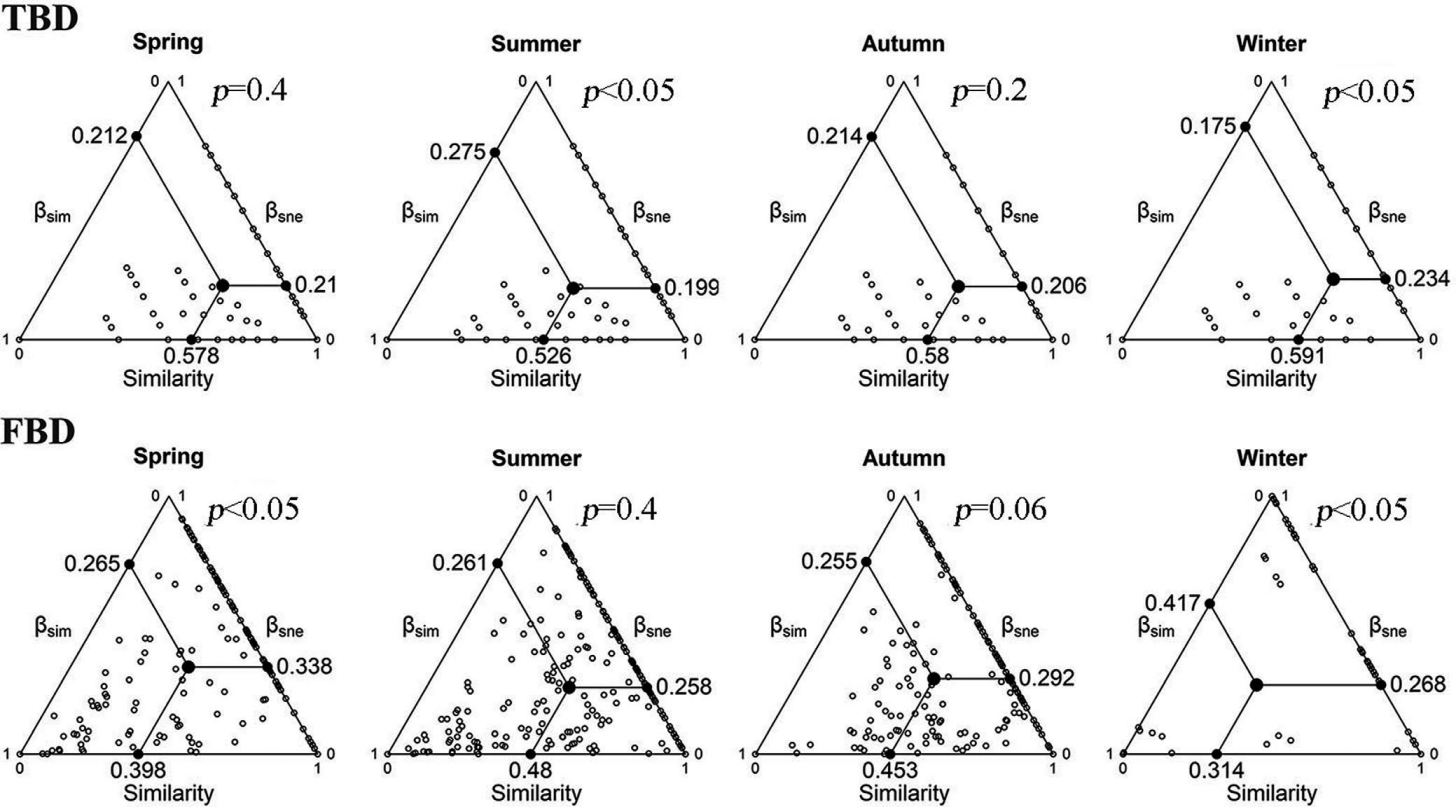
Triangular plots of relationships between beta diversity and two components for pairs of sites in Hongshan Bay considering taxonomic beta diversity (TBD) and functional beta diversity (FBD). Position of each black circle is determined by values of similarity = (1 – beta diversity), turnover (βsim), and nestedness (βsne) matrices. Large solid points in each graph are centroids (black circles), and smaller solid points represent mean values of similarity, turnover (βsim), and nestedness (βsne) components. P‐values represent significance of Wilcoxon signed‐rank tests between turnover and nestedness in each season for TBD and FBD, respectively

Both taxonomic turnover and nestedness and functional turnover and nestedness showed comparable contributions to variations in taxonomic and functional beta diversity across the four seasons. For taxonomic beta diversity (TBD), significant differences in taxonomic turnover and nestedness components were found in summer (*p* < .05) and in winter (*p* < .05), that is, in summer, on average, 58% of species were replaced between pairs of sites and 42% were lost/gained, while in winter, on average, 43% of species were replaced and 57% were lost/gained (Figure 3). In spring and autumn, taxonomic turnover and nestedness showed similar contributions (49% and 51%) to taxonomic beta diversity (*p* > .05). For functional beta diversity (FBD), only winter functional turnover (61%) was significantly higher than functional nestedness (39%), while functional nestedness had a higher or almost equal contribution relative to functional turnover in the other three seasons.

### 3.2 Seasonal variation in taxonomic and functional diversity

**FIGURE 4:**
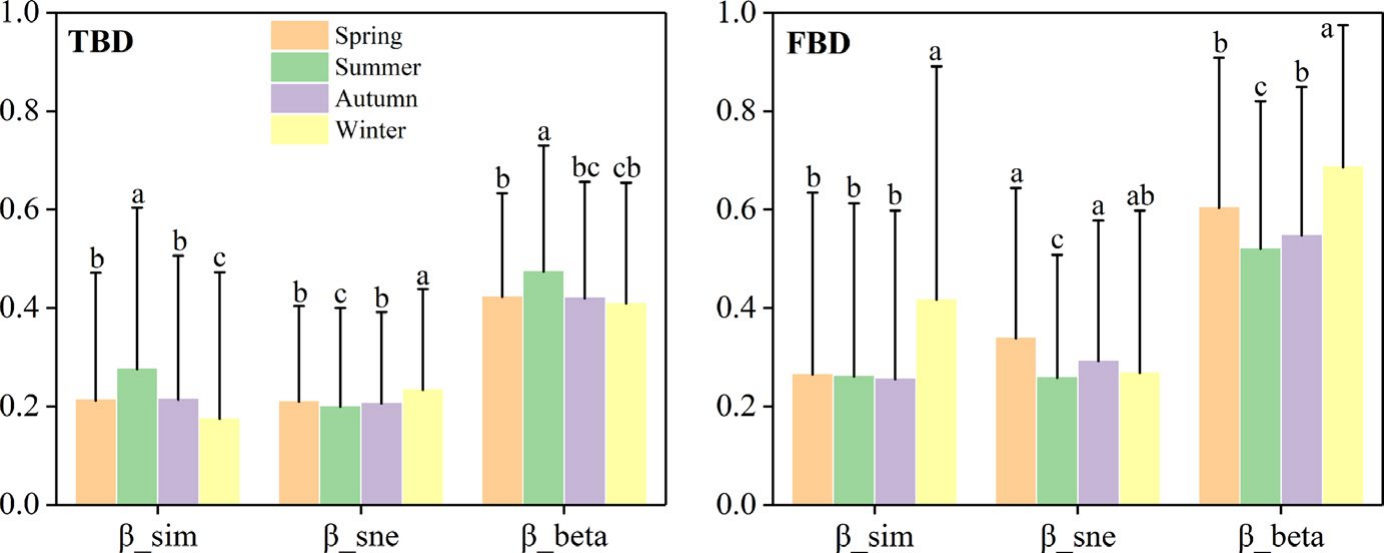
Seasonal changes in submerged macrophyte beta diversity (β_sor), turnover (β_sim), and nestedness (β_sne) across four seasons for taxonomic and functional dissimilarity. Different letters indicate significance of differences in turnover, nestedness, and beta diversity among four seasons determined by Kruskal–Wallis post hoc comparisons at 0.05 significance level

In Hongshan Bay, there were obvious seasonal patterns in taxonomic and functional beta diversity as well as corresponding turnover and nestedness patterns (Figure 4). In general, the functional dissimilarity index (beta diversity, turnover, and nestedness) was higher than the taxonomic dissimilarity index in Hongshan Bay in all seasons. This indicated more divergence in functional community composition than taxonomic composition in the bay. Moreover, taxonomic and functional beta diversity exhibited opposite patterns of seasonal change, with taxonomic beta diversity being highest in summer and lowest in winter, and functional beta diversity being highest in winter and lowest in summer. Taxonomic and functional turnover among pair sites presented similar patterns as taxonomic and functional beta diversity (Figure 4) (i.e., taxonomic turnover was highest in summer and lowest in winter, and functional turnover was highest in winter and lowest in the other three seasons). For the nestedness components, taxonomic nestedness was lowest in summer and highest in winter, while functional nestedness exhibited a lower state in summer.

### 3.3 Role of environmental factors and geographic distance in taxonomic and functional diversity

**FIGURE 5:**
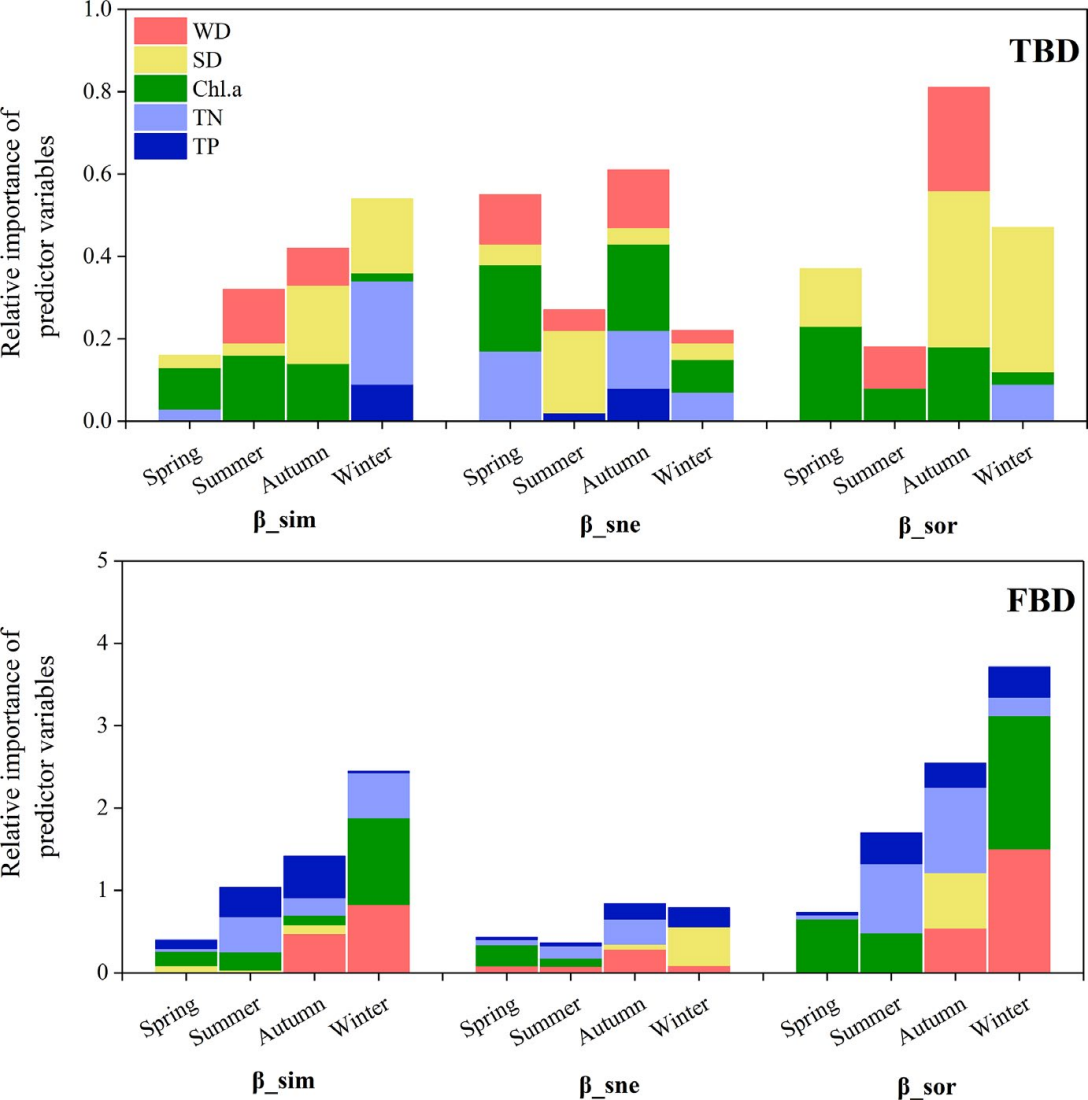
Relative importance of specific predictors for beta diversity, turnover, and nestedness in four seasons determined by summing coefficients of I‐splines from generalized dissimilarity model. TP: total phosphorus; TN: total nitrogen; Chl‐a: phytoplankton chlorophyll‐a; *SD*: water transparency; WD: water depth; total: sum of importance of all environmental predictor variables

Predominantly, across seasons, environmental factors were the main drivers of beta diversity, turnover, and nestedness, regardless of the taxonomic or functional diversity index (Table 1). Only in spring, pure spatial drivers had a similar impact as pure environmental drivers on functional beta diversity and nestedness components (Table 1). Environmental factors better predicted the functional diversity index (i.e., beta diversity, turnover, and nestedness) than the taxonomic diversity index (Figure 5).

**TABLE 1:** Relative importance of environmental factors and geographic distance in taxonomic (TBD) and functional beta diversity (FBD) and corresponding turnover and nestedness components in four seasons. pE: proportion of total variation purely explained by space; pS: proportion of total variation purely explained by environment; shared: proportion of total variation jointly explained by environment and space

		Spring			Summer			Autumn			Winter		
		pE	pS	Shared	pE	pS	Shared	pE	pS	Shared	pE	pS	Shared
TBD	β_sim_	1			0.96	0.01	0.03	1			0.95		
	β_sne_	0.86	0.16		0.99		0.01	1			1		
	β_sor_	0.99	0.01		1			1			1		
FBD	β_sim_	0.96	0.07		1			0.84	0.14	0.03	1		
	β_sne_	0.43	0.50	0.07	0.97		0.03	1			0.82	0.18	
	β_sor_	0.62	0.47		1			0.94	0.03	0.03	1		

Of all predictors, Chl.a and *SD* were the most important environmental drivers for both taxonomic beta diversity and functional beta diversity across seasons (Figure 5, Figures S1 and S2). TP had an important influence on functional beta diversity and its turnover in autumn and winter. Importantly, the rate and magnitude of species and functional turnover or nestedness also changed along environmental gradients (Figures S1 and S2). For example, with increasing Chl.a concentration differences among sites in spring, taxonomic nestedness (β_sne_) gradually increased. However, taxonomic beta diversity and turnover increased sharply when the changes in Chl.a concentration were small. When the Chl.a concentration changed over a certain value, the increase in taxonomic beta diversity and turnover slowed (Figure S1).

## 4 DISCUSSION

We explored seasonal variation in taxonomic and functional beta diversity of submerged macrophytes at a fine scale, as well as the underlying mechanisms responding to environmental factors and geographic distances. Firstly, our results indicated that both nestedness and turnover processes were important drivers of taxonomic and functional dissimilarity among submerged macrophyte communities in Hongshan Bay. Secondly, taxonomic and functional beta diversity and the related turnover and nestedness components showed significant seasonal patterns. Notably, taxonomic and functional beta diversity showed the opposite patterns of change (i.e., taxonomic beta diversity was highest in summer and lowest in winter, while functional beta diversity was lowest in summer and highest in winter). Thirdly, in Hongshan Bay (fine scale), environmental filtering of species and traits was the main process driving spatial variation in submerged macrophytes, rather than dispersal limitations.

Both taxonomic turnover and nestedness as well as functional turnover and nestedness showed comparable contributions to taxonomic and functional beta diversity variations. This does not accord with our first assumption that turnover would be the main component of both taxonomic beta diversity and functional beta diversity. Regarding aquatic plants at the fine scale, this is the first study to observe that turnover and nestedness patterns had equally important effects on beta diversity. Compared to previous research, Boschilia et al. (2016) only observed the prevalence of taxonomic turnover in macrophyte beta diversity at the small scale (five arms of Itaipu Reservoir) due to distinct environmental factors. This may be because there was a wide magnitude of environmental gradients in Hongshan Bay; that id, environmental factors (Chl.a and *SD*) may have had a strong effect on nontolerant species, resulting in a decrease in the number of species from one site to another, thus leading to community dissimilarity nestedness in Hongshan Bay (as seen in Figure 5, Figures S1,S2). Similarly, da Silva et al. (2018) found that higher elevation sites experience more extreme seasonal and daily temperature variations, which may cause nestedness‐related temporal dissimilarity patterns. He et al. (2019) also reported that *Vallisneria natans* is dominantly colonized in sites with high basin slope and moderate–high water depth, indicating that community structure is simpler under extreme environmental conditions (i.e., community assemblages become nested under extreme environments). In the current study, however, we did not consider the impact of species competition and other environmental factors (e.g., sediment nutrition, lake bottom topography) on nestedness patterns. For example, interspecies competition in natural communities can eliminate less competitive species, thereby shaping community structure. Fu, Zhong, Yuan, Xie, et al. (2014) reported that niche differentiation plays a structuring role in macrophyte community assembly.

Our results add novel insights emphasizing the temporal variability of taxonomic and functional beta diversity patterns as well as corresponding turnover and nestedness patterns. We found that taxonomic beta diversity was highest in summer and lowest in winter, while functional beta diversity was highest in winter and lowest in summer. This did not accord with our second hypothesis that both taxonomic beta diversity and functional beta diversity would be low in summer and high in winter. Our results suggested that taxonomic differentiation in community composition was greater in summer than functional differentiation but exhibited the opposite pattern in winter. This may be because submerged macrophytes prospered in summer (e.g., increasing ecological range, wider distribution) but declined in winter (e.g., shrinking spatial distribution), leading to spatial dissimilarity patterns in species and traits among the four seasons. Our results also demonstrated that species identity alone does not provide reliable information about biodiversity patterns (Carvalho et al., 2020), which is important for the protection and management of biodiversity. Thus, the taxonomic and functional characteristics of community organization patterns should be considered comprehensively (Hill et al., 2019; Socolar et al., 2016).

**FIGURE 6:**
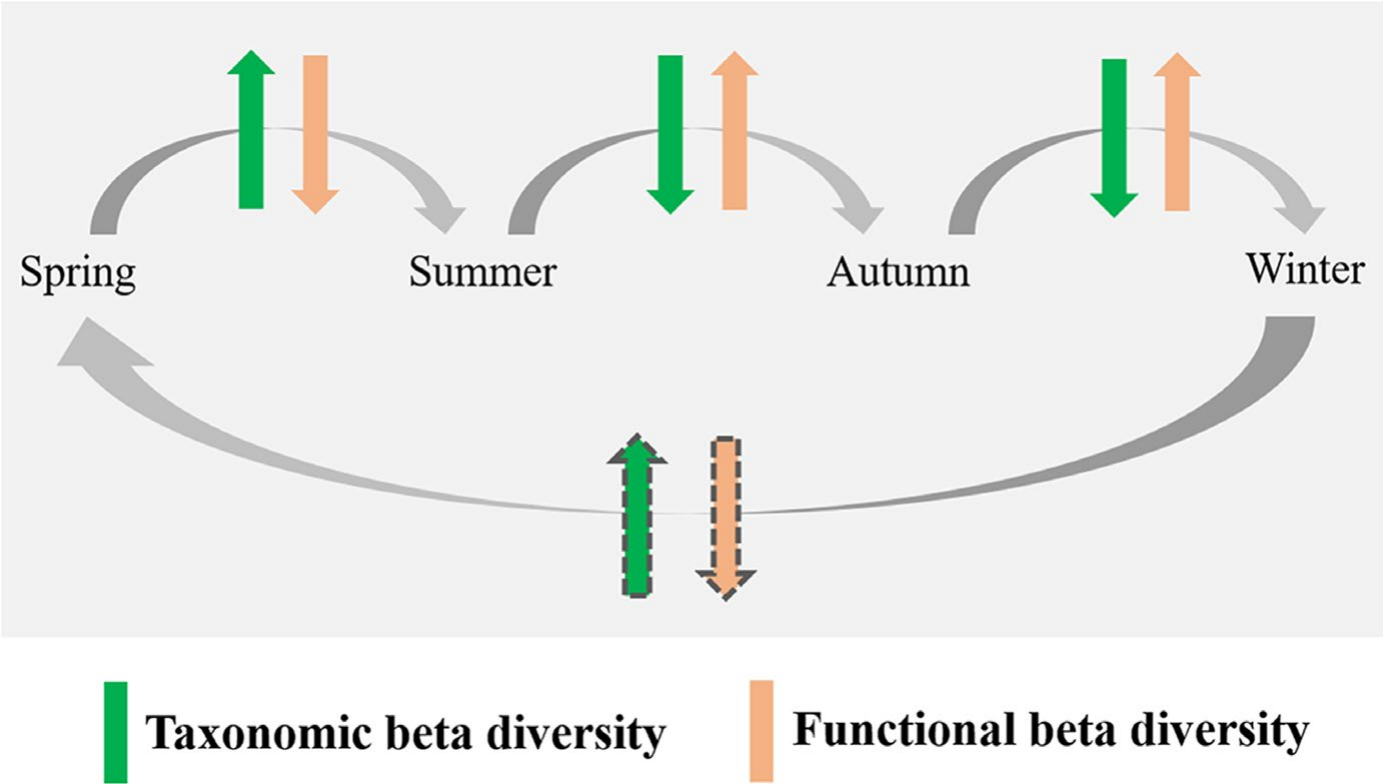
Schematic of variation patterns in taxonomic and functional beta diversity of submerged macrophytes with season in a stable climax community

The submerged macrophyte community in Hongshan Bay has maintained a stable climax community dominated by *Potamogeton maackianus* (>70%) from at least 2011 to 2018 (He et al., 2015; Wang et al., 2020). We found that taxonomic beta diversity gradually increased from spring to summer and decreased from summer to autumn and winter (Figure 4), whereas functional beta diversity demonstrated the opposite change (Figure 4). Thus, the submerged macrophyte beta diversity patterns in Hongshan Bay may show seasonally cyclic changes based on life history to ensure the long‐term stability of the community structure, as shown in Figure 6. Taxonomic beta diversity decreased from summer to winter and increased from winter to summer, whereas functional beta diversity showed the opposite patterns. Similar seasonal patterns (or seasonal cyclical changes) may occur in other stable biological communities, but this requires further research. Thus, for slow population dynamics (e.g., terrestrial forest and grassland communities), single snapshot studies may be suitable to quantify the role of community assemblage drivers at large spatial scales. In contrast, organisms in freshwater ecosystems experience considerable seasonal changes and are more easily affected by water environmental factors. As such, the temporal patterns of spatial variation should be considered over time (Erős et al., 2012).

Beta diversity will produce varied patterns under the different contributions of environmental and geographic distance across various spatial scales (Declerck et al., 2011; Fu, Yuan, Jeppesen, Ge, Li, et al., 2019; Mykrä et al., 2007). At the fine scale, species with different traits can disperse to suitable environments, thus increasing species replacement via species sorting (Gianuca et al., 2017). Compared to geographic distance, environmental factors played a key role in all dissimilarity metrics (taxonomic and functional facets), lending further support to our third hypothesis that environmental filtering more greatly contributes to species and trait turnover. Boschilia et al. (2016) found that high beta diversity (due to spatial turnover) in aquatic macrophyte assemblages is correlated with local‐scale environmental factors. Fu, Yuan, Jeppesen, Ge, Li, et al. (2019) also found that local and regional drivers (e.g., altitude, TN concentration, TP concentration) contribute to structuring species and functional beta diversity patterns (mainly due to species turnover and functional nestedness). However, when environmental factors change dramatically from suitable to extreme conditions, some tolerant species will be screened out to form nested communities (Bevilacqua & Terlizzi, 2020; da Silva et al., 2018). This process (turnover and nestedness driven by environmental factors) is in line with the catastrophic regime shifts that occur in shallow lakes, that is, when the water environment conditions change drastically, the structure, function, and stability of freshwater ecosystems also change (Scheffer & Carpenter, 2003; Scheffer et al., 2001).

## 5 CONCLUSION

In conclusion, we studied seasonal taxonomic and functional beta diversity patterns in submerged macrophyte communities and found that both taxonomic turnover and nestedness as well as functional turnover and nestedness were important drivers of seasonal variations in taxonomic and functional beta diversity. Environmental variables rather than geographic distance dominated spatial variations in species and trait composition of submerged macrophytes across seasons at the fine scale. Using the trait‐based approach to study the variation mechanisms underlying the community structure of submerged macrophytes, we confirmed the importance of niche‐based processes in community assemblage at a fine scale. Moreover, patterns of taxonomic and functional community structure varied across seasons, indicating the importance of considering functional traits over time to study community structure variation. These results provide a reference for future studies on aquatic plant communities, especially in the management and restoration of local habitats.

## CONFLICT OF INTEREST

None declared.

## AUTHOR CONTRIBUTION

**Hao Wang:** Data curation (equal); Investigation (equal); Methodology (equal); Writing‐original draft (equal); Writing‐review & editing (equal). **Hui Fu:** Funding acquisition (equal); Resources (equal); Software (equal); Validation (equal); Visualization (equal); Writing‐original draft (equal); Writing‐review & editing (equal). **Zihao Wen:** Investigation (equal); Writing‐review & editing (equal). **Changbo Yuan:** Investigation (equal); Writing‐review & editing (equal). **Xiaolin Zhang:** Funding acquisition (equal); Project administration (equal); Supervision (equal); Writing‐review & editing (equal). **Leyi Ni:** Project administration (equal); Resources (equal); Supervision (equal); Writing‐review & editing (equal). **Te Cao:** Conceptualization (equal); Funding acquisition (equal); Project administration (equal); Writing‐review & editing (equal).

### DATA AVAILABILITY STATEMENT

Data used in this manuscript were submitted to Dryad and are preliminarily available at https://doi.org/10.5061/dryad.kwh70rz3c.

## Supporting information

Supplementary Material
