# Supplementary material for: Seasonal patterns of taxonomic and functional beta diversity in submerged macrophytes at a fine scale
Source: Ecol Evol. 2021 Jun 24;11(14):9827–36. doi: 10.1002/ece3.7811 (PMC8293774; doi:10.1002/ece3.7811)

**Fig. S1.** Taxonomic beta diversity (βsor), turnover (βsim) and nestedness (βsne) along environmental gradients in four seasons. TP: total phosphorus; TN: total nitrogen; SD: water transparency; Chl.a: phytoplankton chlorophyll a; WD: water depth


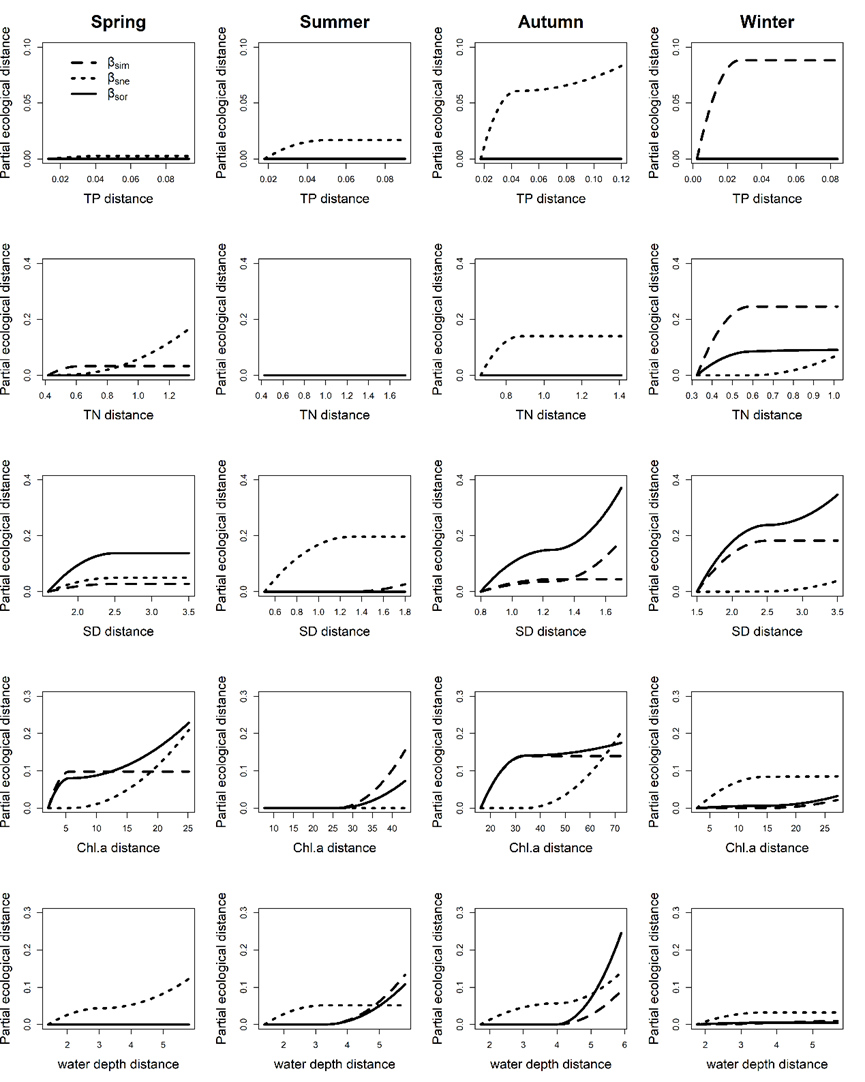


**Fig. S2**. Functional beta diversity (βsor), turnover (βsim) and nestedness (βsne) along environmental gradients in four seasons. TP: total phosphorus; TN: total nitrogen; SD: water transparency; Chl.a: phytoplankton chlorophyll a; WD: water depth


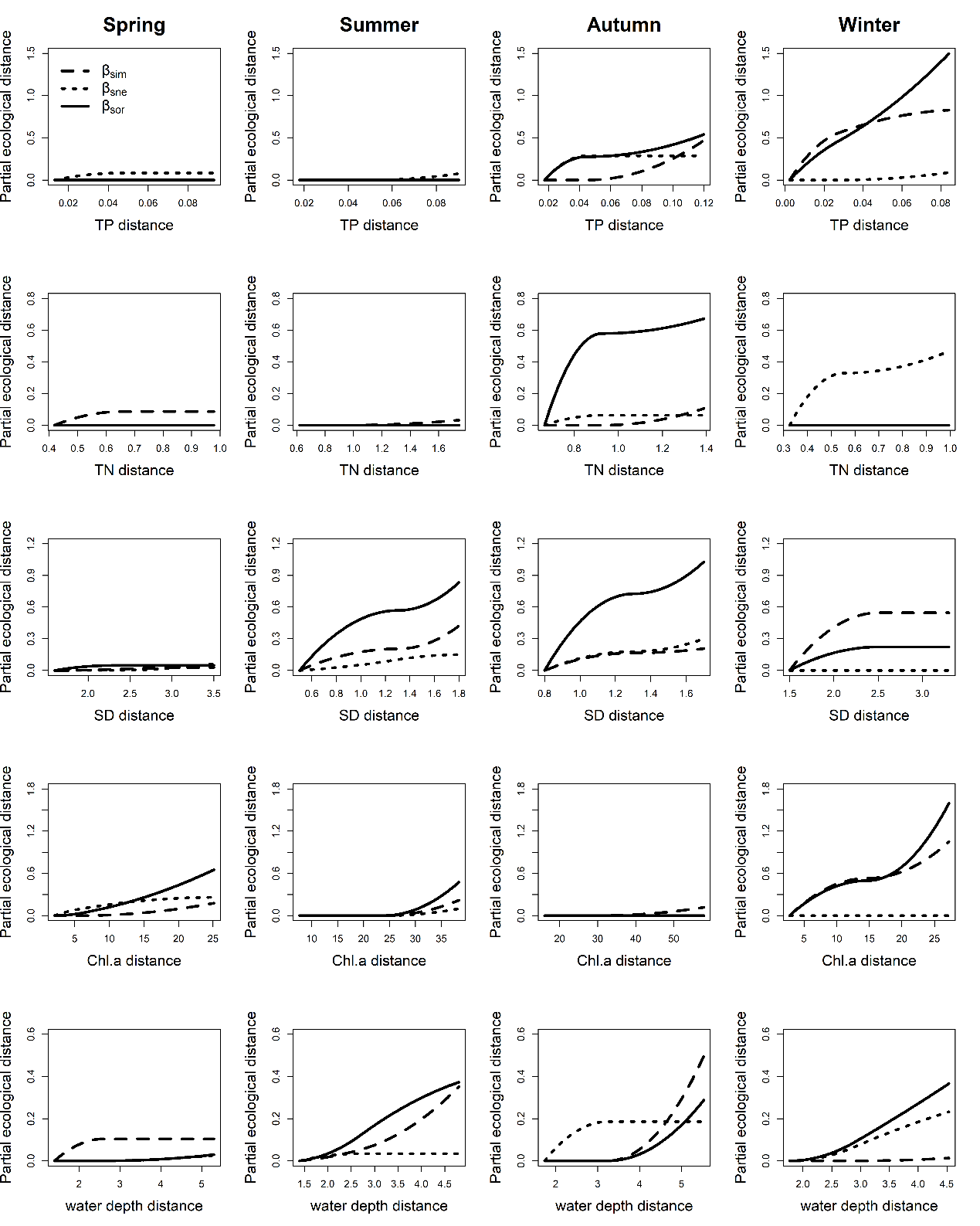

Supplement: Supplementary file 1 — Supplementary Material [file ECE3-11-9827-s001.docx]
